# Meeting Reports

**DOI:** 10.1155/S1463924679000456

**Published:** 1979


### Meeting Reports

### Thirtieth Pittsburgh Conference

The 30th Pittsburgh Conference was held from the 5th to 9th March 1979 at the Convention Center, Cleveland, USA. The theme of the conference was 'Continued Growth in Analytical Chemistry and Applied Spectroscopy through Expanded Communication'. In every sense the 'Cleveburg' conference achieves this aim and provides an excellent opportunity for meeting new people, gaining new interests, and indeed it furthers the communications between users, researchers and instrument companies.

Automation was covered in many of the lecture sessions and also by many exhibitors. However, the general confusion with respect to terminology resulted from the fact that all aspects of automation were not linked together in selected symposia. Two sessions however were devoted to aspects of automation and the papers that were presented in these are discussed here. Since there are no formal publications associated with the meeting, the authors and their affiliations have been indexed here to enable the reader to obtain more details of each paper. Other selected papers presented throughout the five days are also discussed with a brief overview of the instrument exhibition.

Luft [1] described the advantages of discontinuous feedback control for laboratory automation offered through a commercially available system. Feedback control focusses on results and any deviation from a pre-set level causes the control system to correct the process. The disadvantages of continuous control most often provided by computer systems were ennumerated. Discontinuous control utilises a well-tested algorithm. When the controlled variable is far away from the set point the controller operates at high gain and at the appropriate time it automatically switches to a lower adjustable gain. When the set point is reached control may continue or be terminated with or without a delay. Complex control problems can be handled by the controller by considering them as a set of simple problems either in sequence or in parallel. The controller also operates in distributed systems with computers and other devices, and applications described include titrations, gradient and temperature programming and reaction rate studies.

In two papers Sadtler [2] described an instrument used for thermometric titrations and detailed interesting applications. The system, available commercially as the SANDA thermo-titrator, comprises an automated burette, a magnetically stirred Dewar vessel (an adiabetic reaction vessel) and a control module which provides a direct readout of the equivalence volume. For exothermic reactions the response of the thermo-titrator is a linear function of the titrant added. The instrument is unaffected by colour or turbidity and can be operated in slurries. Applications to measure the active surface area of inorganic catalysts were described.

Fletcher [3] described an automated system to assist the technician in the determination of percent non-volatiles. Essentially it comprises an electronic balance interfaced to a programmable calculator. Flexible software enables a range of applications which are easy to use and reduce errors. Implementation of this system has doubled the technicians' output.

Kimbrell [4] described a collaborature test of the Houston Atlas equipment for measuring the total sulphur content in liquid hydro-carbons.

Precision data and modifications made to improve syringe drive and methodologies for high molecular weight sulphur compounds were described. Rogers [5] showed how a microprocessor system had made the development of a laboratory octane number analyzer possible. The conditions in an engine are simulated, fuel/air mixtures injected into a heated chamber and a cool flame generated. The induction time and the intensity of the flame front are measured and once calibrated, the octane number can be printed directly. Self diagnosis packages are also available.

In Arndt's absence, Shauwecker [6] presented a description of the Mettler automated solid liquid extractor. This was described in detail in the *Journal of Automatic Chemistry* 1978, **1**, 28. Considerable manpower savings are possible using this device alone or in association with other modules in the instrument range.

Fernandez [7] described two configurations of the SANDA dissolution rate analyser for research and quality control applications. Comparisons of the automated system was made with typical manual analysis. Applications include drug dissolution rate profiles using UV colorimetric or fluorometric determinations.

The final two papers in this session described complex sample handling problems, handling slurries and acid digestion. Brown [8] described an automated system for preparing and precisely sampling slurries such as whole meals, salad dressings, meats and pastries. The system components are a modified Omni stirrer, an easily machined dispenser tip and valve, and a simple electronic controller. Nitrogen is purged through the apparatus to minimise oxidation damage. A continuous slurry of the homogenised sample is pumped using an inert gas overpressure and sampled discretely. Determinations for moisture, total lipid and fatty acids made using the automated slurry sampler described for replicate samples compare favourably with conventional manual methods.

Knapp [9] described two mechanised methods of decomposing organic traces and discussed their relative merits. The first of these mechanises a wet chemical oxidation using any of the normal wet ashing reactions and operates quasi continuously to a preprogrammed sequence of time and temperature. In the second procedure samples are incinerated in the presence of pure oxygen within a quartz chamber. The volatile components are driven off and the involatiles which remain are solubilised by refluxing with nitric acid. Two samples an hour can be handled by the well controlled system; all steps are processor controlled.

The second symposium devoted to laboratory automation centred in the main part on electro-chemical and spectroscopic methods. Burrow [10] discussed the need to monitor the purity of silicon tetrachloride and also described the injection loop and analytical methodology used to prevent contamination of the product and to monitor the chemicals involved. The method requires the reaction of $SiCl_4$ with magnettion powder to generate HCl, distillation of the excess $SiCl_4$ and measurement of the HCl produced spectro photometrically. Levels of 1ppm of HCl can be measured and on-line applications have been considered. Cooley [11] described an automated sampler system and discussed its application with a commercial scanning polarograph. It is flexible and overcomes many of the problems inherent in models such as the Technicon SOLIDprep sampler. The sampler which is interfaced to an Intel 8080 microprocessor controller dissolves, mixes and dispenses the sample from the same cup. Twenty five samples can be analysed within a complete cycle and stirring is facilitated by adding a magnetic stirrer to each cup. Unattended operation overnight greatly increases the sample through-put of the analysis system described.

Das Gupta [12] discussed the theory and application of hydrodynamic voltametry to online monitoring of environmental pollution and chemical process control. Maintaining this theme Fleet [13] discussed the relative merits of potentiometric, polarographic, voltametric and amperometric methods for determining cyanide.

Stockwell [14] presented an overview of the introduction of automation and computing techniques to the survey of cigarettes to determine their relative deliveries of tar, nicotine and carbon monoxide. These techniques were applied in the experimental design stage to sampling, preparation, measurement and the reporting stages of the procedure. Discrete, continuous flow and on-line analytical techniques are combined to maximise the output of the survey.

Ghadimi [15] described the design of a new generation continuous flow analyser with particular emphasis on the optical configuration; significant improvements were claimed for this. Sample cells with 75, 25 and 15 mm pathlength can be easily accommodated. This is a reliable, low maintenance continuous on-line colorimetric cell which also has a low dead volume making it quick and easy to transfer from one analyte concentration to another. The analysers have been used to measure a number of parameters such as free chlorine, total chlorine, hardness, nitrate, phosphate, iron and copper in water samples.

Stewart [17] discussed the use of flow injection analysis techniques for automated titrations. He presented a completely automated system which can be quickly implemented using readily available components. A simple, easy-to-construct colorimeter and a controlled dispersion mixing chamber were also described. The effect of variable sample volume on analytical performance was also discussed.

A modular syringe drive mechanism, capable of carrying out multimixing experiments using reagent sample loops, was described in detail by Holler [18]. The drive mechanism is controlled through an opto-electronic controller. Rapid reaction rate experiments were used to illustrate the flexibility and performance of the device.

In the final paper of the session Matsuzaki [19] discussed the performance of the Dohrmann microcoulometric titration system and its application in the single boat inlet mode for the analysis of chlorine in hydraulic fluid and lubricating oils. Interference problems and their solutions were also described.

Other aspects of automation were considered in other symposia sessions. Of these, many centred on aspects of microprocessor control and applications. Several papers were presented by commercial companies who gave specific details of their new product lines. Details of this latter group are available directly from the instrument companies' representatives in individual countries. Varian presented details of the instrument required for coupling the output of an HPLC effluent into a gas chromatographic column and applications. The instrumentation was also demonstrated in the exhibition area. The power of this approach for solving analytical problems is realised by sample throughput, use of the combined technique to solve separation problems, two independant retention times to aid component identification and the use of combined detection techniques for specific identification.

Silverman [20] described a version of a gas chromatograph heat cutting system equipped with a cold trap and heater system so that a condensed portion of the sample can be reinjected onto a second column to expand the analysis. Perkin-Elmer introduced a new microprocessor controlled auto-sampler which provides increased flexibility, ease of operation and two-way communication between the sampler, chromatograph and data system. The samples are forced through a needle as a fluid rather than being pulled up by vacuum and this eliminates any fractionation in the sampling process. Carlo Erba discussed the combination of their capillary gas chromatograph,which is finding much favour in Europe,with an automated head space analyser.

Pope [21] described a prototype sampling technique to 'grab' and seal up to 26 individual portions of environmental and drinking water to analyse for trace organics. The water sample is only exposed to Teflon and glass components and it can be refrigerated to 4°C for storage. Dahnke [22] discussed the automation of a microprocessor controlled ion chromatograph which is one of the Dionex range. This was also demonstrated in the exhibition area. The chromatograph is interfaced to a Gilson auto-sampler and to an IBM 1800 computer system which when suitably programmed allows the system to be operated overnight without operator intervention

Kuehl [23] described an automated interface for sampling the effluent from an HPLC column and directing it to a Fourier transform infrared instrument. The majority of the solvent from the column is evaporated in a heated tube. Samples, up to 31 from each chromatograph, are retained in a 5 mm cup containing KCl on a frittered filter. In two papers authors from Technicon described the application of their fast HPLC system in the estimation of vitamins and a general overview of the application of pre- and post-column reactions in HPLC analysis.

In addition to the papers discussed above, a symposium session was devoted to Laboratory Data Management. Four papers were presented in the session which dealt with particular aspects of the problem as applied to the speakers' own laboratory interests. Sample inventory, management and quality control was the theme of Cupps [24], whilst Madsen [25] described the problem of paperwork in a regulatory agency. Problems specific to the pharmaceutical industry were detailed by Johnson[26]. Progress towards a computerised laboratory was the subject of Fletcher's [27] interesting talk.

Whilst the lectures provide a unique forum for discussions, the most valuable aspect of this conference is without doubt the exhibition. Each year the size and scope increases and over 900 booths representing 350 companies made up the exhibition this year.

Providing details of each of these is obviously a mammoth task which cannot be covered in these pages. In future issues, details of products released at the conference will be covered but remarks here will be restricted to initial reactions. Microprocessors are now common place; indeed many companies are on their second generation of instruments. How they are applied varies both between companies and indeed within company ranges. The Du Pont thermal analysis system seems to offer good facilities allowing measurement, analysis and reporting stages to be carried out in parallel. It offers flexibility and improved sample throughput. In contrast the company's liquid chromatograph does not seem so well designed.

Hewlett Packard's new range of gas chromatographs overcomes many of the limitations of earlier models, although the sampler itself could still be improved. The Varian segmented tray approach offers more flexibility. Hewlett Packard's range can be expanded to meet changing needs and experience gained in the gas chromatograph has also aided the development of a new range of liquid chromatographs. Whilst the detector systems need to be proved, in practice the flexibility and control offered by the range is potentially valuable. Much interest also centred on Perkin-Elmer's infrared spectrometer and data handling system originally launched at the FACSS meeting.

There is a considerable shift towards the use of integrated visual display units for data presentation. It is very surprising, in view of the fact that these devices are so common place, (commercial airlines operate almost exclusively using such units), that instrument manufacturers should begin by redesigning these units. Units on display ranged from small screen modules, which are difficult to read, to the use of coloured displays for simple numerical output. Some attempt at standardisation would have seemed sensible. However it seems that manufacturers have resolved to the 'gimmickry' approach rather than the practical one.

Surely the purchasers of expensive and sophisticated instruments deserve better than this. One very interesting application of these units was presented by Videochart [28]. The recorder displays complete zones of data and allows the operator flexibility to examine individual segments, to normalise data and to add or subtract a similarly recorded data output. The simple to use device will find many applications for the development of analytical procedures.

*Peter B. Stockwell*

BIBLIOGRAPHY

[1] Discontinuous feedback control in laboratory automation. Luft, L., Luft Instruments Inc, Hillside Road, Lincoln, MA 01773, U.S.A.

[2] Thermometric titration system using an automatic digital end point indicator. Sadtler, P., Sanda Inc, 4005 Gipsy Lane, Philadelphia, PA 19144, U.S.A.

[3] Automation of the percent non-volatile analysis. Fletcher, P., PPG Industries, Coating and Resins Research Center, Allison Park, Pennsylvania, 15101, U.S.A.

[4] Results of an inter laboratory co-operative test on total sulphur in liquid hydrocarbons. Kimbrell, C., Houston Atlas Inc, 9441 Baythorne Drive, Houston, Texas 77041, U.S.A.

[5] Microprocessor controlled laboratory octane analyzer. Rogers, L., Foxboro Analytical, Arcas Center, 10707 Haddington Drive, Houston, Texas 77043, U.S.A.

[6] Automated solid-liquid extraction. Arndt, R., Mettler Instrumente, AG, CH-8606 Greifensee, Switzerland.

[7] SANDA: dissolution tool of the future. Fernandez, J, Technicon Industrial Systems, Tarrytown, New York 10591, U.S.A.

[8] Inexpensive system for rapid precise slurry sampling. Brown, J., U.S. Dept. of Agriculture, Nutrition Composition Laboratory, Agriculture Research, Beltsville, Maryland 20705, U.S.A.

[9] The application of two mechanised methods for the decomposition of organic materials to trace analysis. Knapp, G., Institute for General Chemistry, Technical University Graz, Technikerstrabe 4, A-8010, Graz, Austria.

[10] On stream analysis $SiCL_4$ during the production of solar grade silicon. Burrow, G., Westinghouse R & D Center, Pittsburgh, PA 15235, U.S.A.

[11] An automated scanning polarograph for solution labile compounds. Cooley, R, Analytical development, Eli Lilly and Co. P.O. Box 618, Indianapolis, Indiana 46206, U.S.A.

[12] Hydrodynamic voltammetry-theory and application to chemical analysis. Das Gupta, S., HSA Reactors Ltd., 17, 44 Fasken Drive, Rexdale, Ontario, Canada M9W 5M8.

[13] An evaluation of continuous monitoring procedures for cyanide. Fleet, B., *ibid.*

[14] Automatic large scale routine analysis of cigarette smoke. Stockwell, P., Laboratory of the Government Chemist, Cornwall House, Stamford Street, London SE1 9NQ.

[15] CFA-200, a new automated continuous flow instrument. Ghadini, R., Peerless Electronic Research Corporation, 2000 Shames Drive, Westbury, New York 11590, U.S.A.

[16] A new on-line colorimetric analyzer for water and wastewater Clemens, R., Hack Chemical Company, Center of Applied Analytical Chemistry, P.O. Box 389, Loveland, Colorado 80537, U.S.A.

[17] Automated titrations: the use of automated flow injection analysis for the titration of discrete samples. Steward, K., as reference [8].

[18] Versatile syringe drive module for rapid mixing methods. Holler, F., Dept. of Chemistry, University of Kentucky 40506 U.S.A.

[19] Determination of trace amount of chlorine in petroleum products by combustion and microcoulometric method. Matsuzaki, A., Central Technical Research Laboratory, Nippon Oil Co. Ltd., 8, Chidori-Cho, Naku-Ku, Yokahama, 231 Japan.

[20] Automated GC heart cut analyses. Silverman, H., Hewlett Packard Co., R & D Center, Route 21, Avondale,PA19311, USA.

[21] An automatic sampler for trace organics in water. Pope, J., US Environmental Protection Agency, College Station Road, Athens, Georgia 30605, U.S.A.

[22] A simple automated ion chromatography system, Dahnke, K., Phillips Petroleum Co., 263 Research Buildings, Bartlesville, Oklahoma 74004, U.S.A.

[23] Automated interface between a liquid chromatograph and a fourier transform infrared spectrometer. Kuehl, D., Dept of Chemistry, Ohio University, Athens, Ohio 45701, U.S.A.

[24] Sample and inventory management and quality control. Cupps, J., Gulf Oil Chemicals, Baytown, Texas, 77520, U.S.A.

[25] The paperwork problem of a regulatory agency laboratory. Madsen, F., OSHA Analytical Laboratory, 390 Nakara Way, Salt Lake City, Utah 84108, U.S.A.

[26] Laboratory data management in the pharmaceutical industry. Johnson, R., Control Laboratories, The Upjohn Company, Kalamazoo, Michigan 49001, U.S.A.

[27] Laboratory systems management -progress toward the computerized laboratory. Fletcher, P., as reference [3].

[28] Videochart recorder, P.O. Box 162, Glastonbury, Connetticut 06023, U.S.A.

### Automation in the measurement of corrosion

A meeting on the above topic organized by the Automatic Methods Group of the Chemical Society, Analytical Division was held at the University of Southampton on 2nd March 1979.

Three papers were presented ranging from the fundamental aspects of corrosion, through experimental monitoring of corrosion in high temperature, high pressure boilers to the experience gained in running a computer controlled chemical monitoring system for corrosion control in a nuclear power station. The first paper, given by Alan Bewik from Southampton University, took us expertly through the fundamentals of corrosion. The thermodynamic and kinetic feasabilities of the various anodic and cathodic reactions associated with corrosion were discussed and the processes involved in metal passivation were clearly expounded. Geoff Mann of the Central Electricity Research Laboratory, Leatherhead then discussed the progress being made in simulating the corrosion occuring in high temperature (360°C), high pressure (2600 psi) water boilers, using a specially constructed experimental autoclave within which test specimens could be subjected to a variety of temperatures, pressures and water compositions. A specially devised analytical system was described which enabled such parameters as temperature, pressure, oxygen content of the feed water, pH and hydrogen content of the effluent to be continuously monitored. A number of the analytical techniques described had been specifically developed for this work at C.E.R.L.

Finally, George Bown from Hinkley Point Nuclear Power Station (CEGB) described a computer controlled chemical monitoring system which enabled plant engineers to maintain the environment of the reactors and boilers in order that the ideas expressed by the first two speakers could be put into effect. To do this a system of 25 gas sampling points and 51 water sampling lines had been installed to feed samples to a battery of analysers under the control of a PDP-11/05 computer. This enabled excursions outside pre-set limits to be rapidly communicated to the appropriate engineer, as well as providing daily statistical summaries.

The progression of ideas expounded by the three speakers made for a well thought-out and logically presented meeting much appreciated by the regretably small audience.

*C.J. Jackson*

